# Geological surface reconstruction from 3D point clouds

**DOI:** 10.1016/j.mex.2021.101398

**Published:** 2021-05-26

**Authors:** Cristina Serazio, Marco Tamburini, Francesca Verga, Stefano Berrone

**Affiliations:** a Department of Environment, Land and Infrastructure Engineering, Politecnico di Torino, Corso Duca degli Abruzzi 24, Torino 10129, Italy; b Department of Mathematical Sciences “Giuseppe Luigi Lagrange”, Politecnico di Torino, Corso duca Degli Abruzzi 24, Torino 10129, Italy; c Digitmode srl, Via Cola di Rienzo 4/1, Milano 20144, Italy; d Memeber of INdAM-GNCS, Italy

**Keywords:** Geological surface, Best fitting plane, Concave hull, Delaunay Triangulation

## Abstract

The numerical simulation of phenomena such as subsurface fluid flow or rock deformations are based on geological models, where volumes are typically defined through stratigraphic surfaces and faults, which constitute the geometric constraints, and then discretized into blocks to which relevant petrophysical or stress-strain properties are assigned.

This paper illustrates the process by which it is possible to reconstruct the triangulation of 3D geological surfaces assigned as point clouds. These geological surfaces can then be used in codes dedicated to volume discretization to generate models of underground rocks.

The method comprises the following:

- Characterization of the best fitting plane and identification of the *concave hull* of the point cloud which is projected on it

- Triangulation of the point cloud on the plane, constrained to the *Planar Straight Line Graph* constituted by the *concave hull*

The algorithm, implemented in *C*++, depends exclusively on two parameters (nDig, maxCut) which allow one to easily evaluate the optimal refinement level of the hull on a case by case basis.

## Graphical abstract

**Figure:**
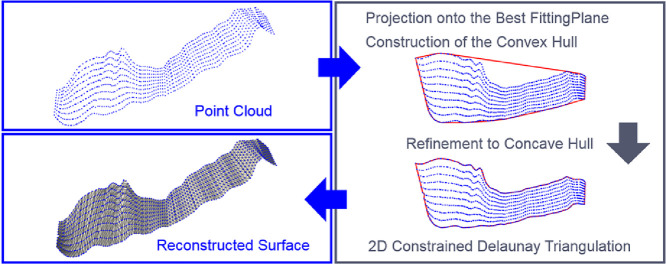

**Table:** Specifications Table

Subject Area	Earth and Planetary Sciences
More specific subject area	*Mesh Generation for Geological Applications*
Method name	*Geological Surface Reconstruction through the constrained Delaunay triangulation of the Planar Straight Line Graph defined by the concave hull segments*
Name and reference of original method	*A New Concave Hull Algorithm and Concaveness Measure for n-dimensional Datasets from*[1]*and Crossing Algorithm from ptinpoly.cpp published in*[2]
Resource availability	*Triangle Library:*https://www.cs.cmu.edu/~quake/triangle.html *Eigen Library*: https://eigen.tuxfamily.org/index.php?title=Main_Page

## Method details

Historically, the process of generating numerical grids for geological applications is constrained to stratigraphic and fault surfaces. In fact, they are the main spatial elements that guide the zoning process of the model as well as its numerical discretization.

The paper describes a method developed to reconstruct these surfaces, represented in the space as generic point coordinates. Classes and functions are written in *C*++ using the C library *Triangle* [3,[4], [10], which operates on the plane. As an output it can provide a *Delaunay* triangulation constrained to a *Planar Straight Line Graph*, which, in this case, is defined by the *concave hull* of the point cloud. The *Eigen* library [5], dedicated to linear algebra, was used for linear algebra operations.

**Fig. 1:**
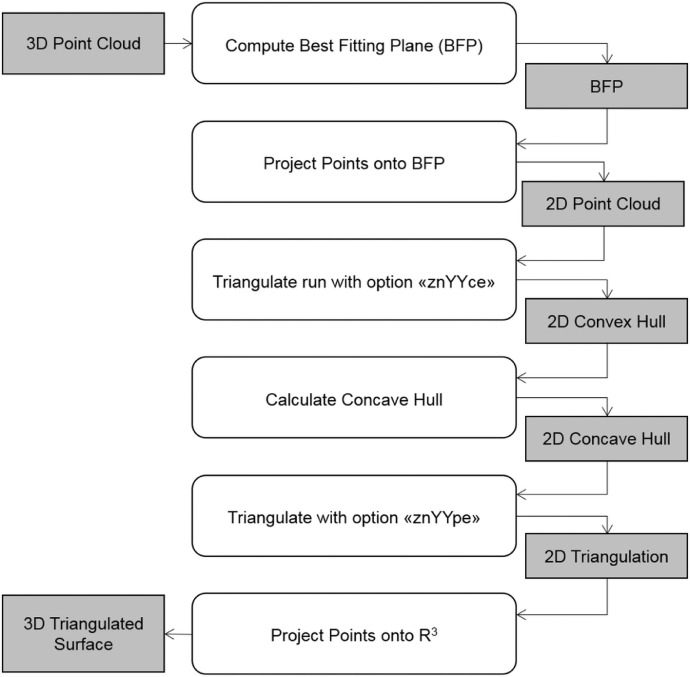
Flowchart of the geological surface reconstruction method.

The main steps of the methodology are schematized in Fig. 1 and described in detail in the sections which follow.

## Best fitting plane projection

**Fig. 2:**
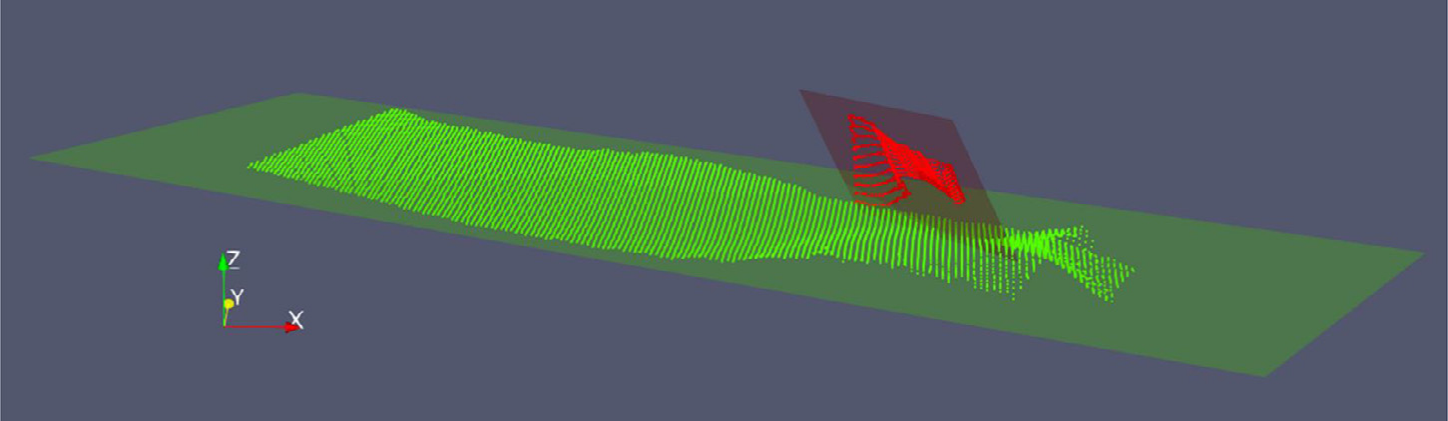
Example of point clouds representing a Stratigraphic Surface (green) approximated with an xy-plane and a Fault Surface (red) with the corresponding Best Fitting Plane.

As already mentioned, the surfaces to be elaborated can be classified in stratigraphic and fault surfaces. If referring to a right-handed Cartesian coordinate system xyz where the z axis is vertically oriented, the stratigraphic surfaces are mainly orthogonal to the z direction, while faults are typically sub-vertical but do not have an orientation in space that can be determined *a priori*. As an example, the clouds of a stratigraphic surface (green) and of a fault surface (red) are shown in Fig. 2.

**Fig. 3:**
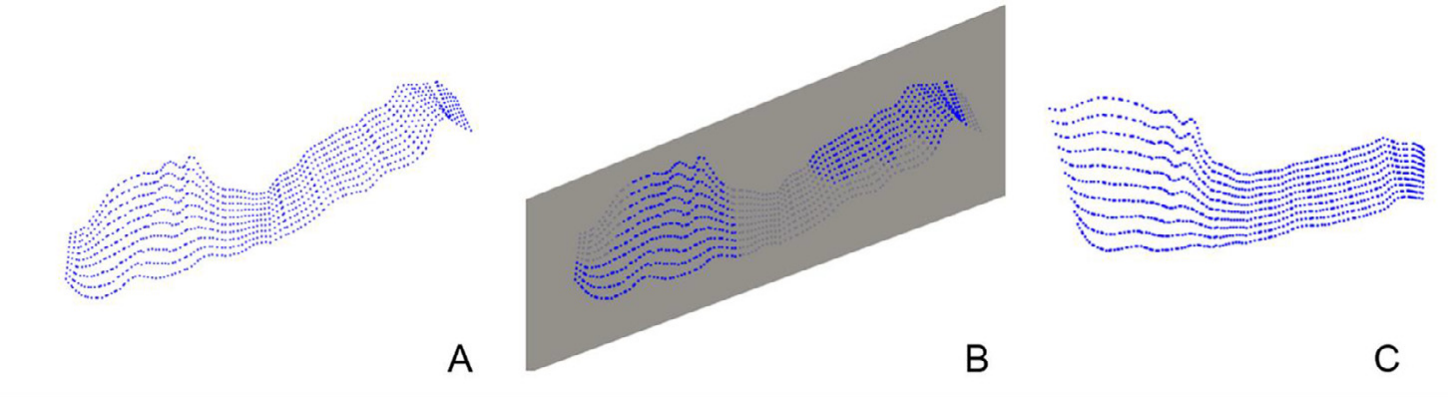
(A) Point cloud, representative of a fault, in the space. (B) Plane (BFP in gray) corresponding to the two main components of the matrix consisting of the coordinates of the points of the cloud. (C) Projection of the point cloud on the BFP.

Therefore, the xy-plane is a potential approximation plane for a stratigraphic surface, whereas, for a fault surface, it is necessary to calculate the principal components of the matrix consisting of the coordinates of the points of the cloud (X), determine the plane identified by them and project the points on it. The detailed procedure is the following: the point coordinates are expressed respect to a Cartesian reference system with origin in the barycenter of the cloud itself and the corresponding matrix $X\in R^{3\;\times \;N\;\text{points}}$ is constructed. The eigenvalue matrix $(D\in R^{3\;\times \;3}$) and the corresponding eigenvector matrix ($V\in R^{3\;\times \;3}$) of $XX^{T}$ were calculated using the Eigen library, then the projections were made on the plane identified by the first two components of the orthonormal basis obtained from V and corresponding to the two larger eigenvalues in D. The related functions are shown in Box 1. Fig. 3 shows, by way of example, a cloud of points in space (Fig. 3A), the calculated BFP (Fig. 3B) and the projection of the points on it (Fig. 3C).

## Hull “digging”

Once the reconstruction problem of the surfaces was brought back to a triangulation on the plane (BFP), the potential of the *Triangle* library was exploited, which implements a stable and efficient Delaunay triangulation algorithm and numerous refinement options. The syntax used for triangulation, without the imposition of any constraints, is shown in Box 2, where the triangleIn and triangleOut structures are the input and output of the routine, respectively. Both are *struct* of the triangulateio type (see Supplementary Material section for details), whose declaration and definition can be found in the files triangle.h and triangle.c of the Triangle library.

**Fig. 4:**
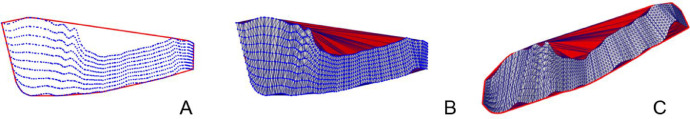
(A) BFP projection of the point cloud and corresponding *convex hull* (red). (B) Triangulation of the BFP point cloud projection with boundary nodes identified by the *convex hull*. (C) 3D space projection of the triangulation. The red area highlights where the triangulation is unsatisfactory due to inadequate identification of the boundary nodes and the polygon that encloses the BFP point cloud projection.

However, if not constrained to the boundary nodes, the point cloud triangulation on the *convex hull* might lead to a poor quality discretization when projected back in the space. This is the reason why a series of triangles (red area of Fig. 4) can be observed, which are incorrectly defined because they connect points that should be tagged as boundary nodes. As a consequence, the triangulation of the fault surfaces in space shows a series of erroneous “folds”.

**Fig. 5:**
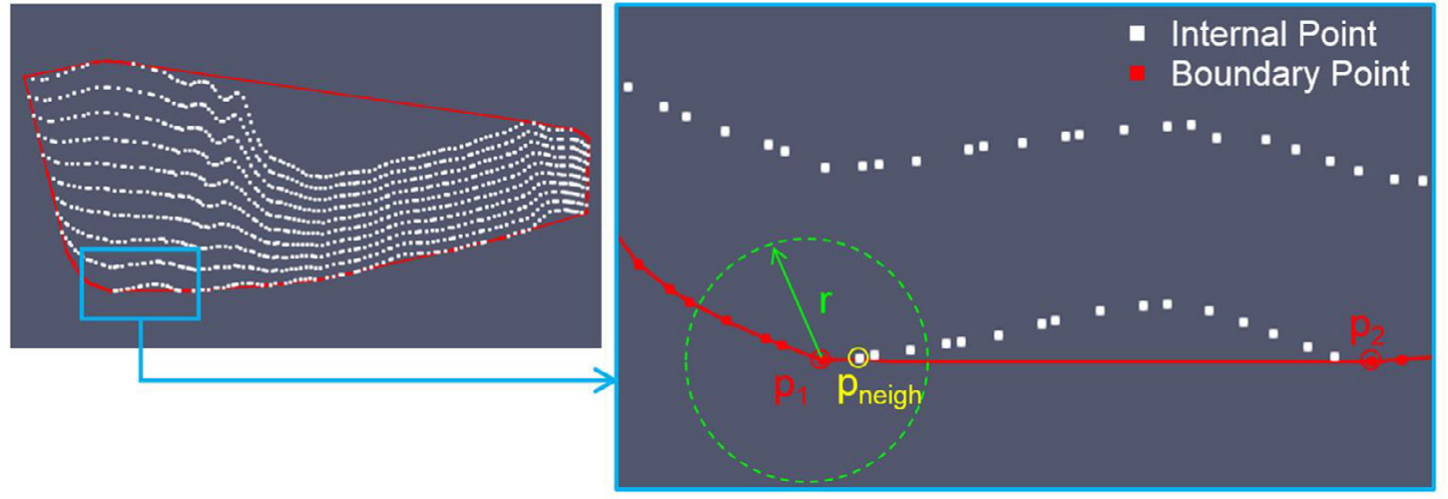
Sketch of the exploration step of the “digging” algorithm: nodes classified as internal in white, nodes and segments belonging to the boundary polygon in red. In a hypothetical iteration, the p_1_p_2_ segment is tested. In particular, the p_1_-neighborhood is defined by the circumference with radius r and center p_1_ and the algorithm searches for internal nodes that fall within it.

Therefore, the need to more accurately identify the polygon that describes the boundary of the BFP projection of the point cloud, i.e. the *concave hull*, became evident. Routines based on the concept of α‐ shapes, available in the CGAL library [6,7], were preliminary tested, but unsatisfactory results were obtained due to the different concavities characterizing the point cloud boundary. For this reason, taking as a reference the “dig criterion” of the *convex hull* introduced by Park and Oh [1], we implemented a variant of the algorithm that is able to optimize the exploration of both the extension and the shape of the neighborhood (for the search of possible boundary points). The algorithm is based on the idea of incrementally “digging” the *convex hull* by iteratively adding a node previously classified as internal to the boundary polygon. If the node passes the admissibility test, it is inserted into the appropriate position and the existing segment of the polygon is “broken”. Fig. 5 shows the exploration scheme of the “digging” algorithm. The starting polygon of the algorithm is the *convex hull* obtained as the output of *Triangle* and the depth to which the hull is “excavated” is determined by the parameter indicated with nDig ∈ [0,1]: it defines the width of the neighborhood exploration through the relation: r=edgeLength*dig. The neighborhood to explore is wider when the dig parameter approaches 1. In a hypothetical iteration of the algorithm, the edge p_1_p_2_ of the current boundary polygon is tested. In particular, the p_1_-neighborhood is defined by the circumference with radius r and center p_1_ and the algorithm searches for internal nodes that fall within it. In Fig. 5, p_neigh_ (yellow) is selected and it is tested, i.e. it is verified that the trial boundary edges p_1_p_neigh_ and p_neigh_p_2_ do not intersect any edge of the current boundary polygon and that no internal points fall in the triangle p_1_p_neigh_p_2_. If the point p_neigh_ passes the test, then it is tagged as boundary node and the p_1_p_2_ edge is “broken”. The Box 3 reports the pseudo-code instructions of the updateHullDig function and Table 1 summarizes the main variables and type and provides their description. The additional routines which are called are reported in Box 7-Box 17 (see the Supplementary Material section) for completeness.

**Table 1:** Description of the main variables defined in the pseudo-code.

**Fig. 6:**
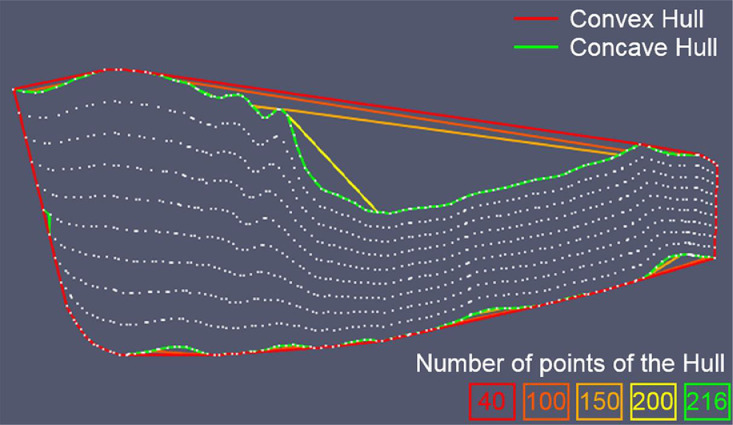
Example of the evolution of the “digging” algorithm of the *convex hull* (red). Initially, the boundary polygon consists of 40 nodes only, iterations at 100, 150, 200 nodes and the final boundary, called *concave hull*, consisting of 216 nodes (green).

Fig. 6 shows an example of evolution of the "digging" algorithm. The initial *convex hull* (in red) and four *concave hulls* of increasing number of nodes (from orange to yellow) are represented. The result is the green boundary.

In the development of the method, some distinctions had to be made between fault surfaces and stratigraphic surfaces due to the intrinsic characteristics of the latter. First, we observed that the calculation of the BFP was not needed as, generally, a BFP can be approximated by a xy-plane of a right-handed Cartesian coordinate system with z as the vertical axis. Secondly, the point clouds that we have to process are typically surfaces deriving from geological modeling and sampled with a constant step on the plane. It follows that the points, once projected onto the plane, have a regular distribution and, in particular, they are aligned with the coordinated axes or with the diagonals, falling within the limit configurations that make the implemented *crossing* algorithm less accurate [8]. Indeed, one of the implemented core function belongs to the family of strategies called *point-in-polygon*, whose criticalities are extensively tested in [9].

**Fig. 7:**
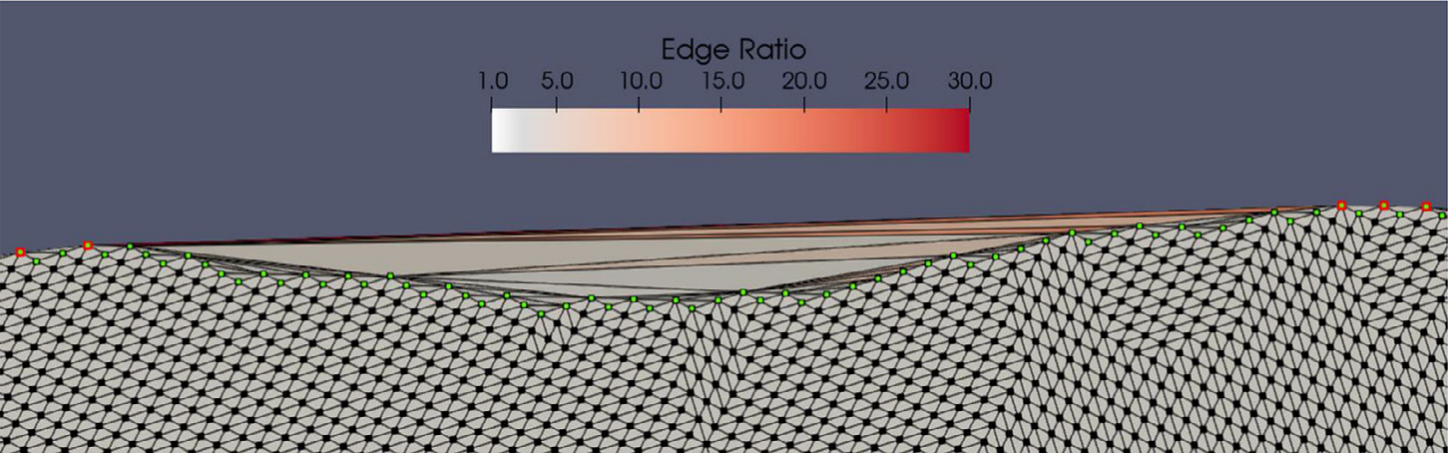
Portion of the point cloud relative to a stratigraphic surface projected on the xy-plane. The colormap refers to the quality of the triangulation (edge ratio) constrained to the *convex hull* (nodes in red). The triangles that deviate from the average value (~ 1.2) are the triangles that should be removed from the triangulation, as they are external to the polygon (nodes in green) that describes the boundary of the cloud.

In order to overcome these limitations, we decided to exploit the information deriving from the preliminary triangulation, i.e. the triangulation delimited by the *convex hull*, so as to apply a different “digging” strategy. We observed that, since the sampling is regular, the quality of the mesh, measured as *edge ratio* (i.e. ratio between the maximum edge length and the minimum edge length of each triangle of the grid), is roughly constant over the whole domain. Triangles characterized by edge ratio higher than average are found exclusively close to the boundary polygon where it is known that the *convex hull* is not adequate to describe the profile of the point cloud. As an example, Fig. 7 shows a portion of the triangulation of the point cloud of a stratigraphic surface projected onto the xy-plane, delimited by the *convex hull* (nodes in red). The color map describes the trend of the edge ratio. Triangles that deviate from the average value (approximately equal to 1.2) are just the triangles that should be excluded from the triangulation because they are external to the polygon that describes the boundary of the projected cloud (nodes in green).

**Fig. 8:**
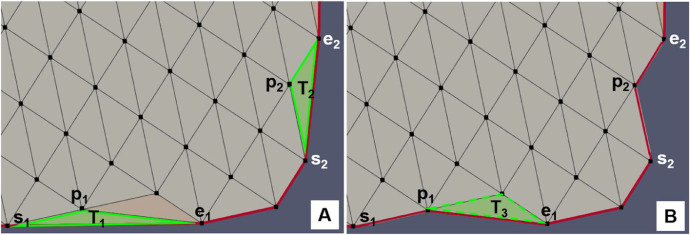
Sketch of the steps for the boundary polygon (in red) refinement algorithm which includes two cycles. The first is the initialization cycle of the outlierTriangles (T_1_, T_2,_ T_3_) and starterTriangles lists (T_1_ T_2_ in green) in A. In the second cycle the starterTriangles list is updated iteratively by deleting the triangles that were removed from the triangulation and by adding the outlierTriangles that have an edge in common with the updated edge polygon (T_3_ in B) at the end of the list.

Based on such considerations we decided to calculate the mean (t.mu) and the standard deviation (t.sigma) of the triangle edge length distribution of the initial discretization. At the same time, two lists of triangles were populated: the first, named outlierTriangles, contains all the triangles that have at least one edge of length greater than thresholdLength=t.mu+maxCut*t.sigma, where $\text{maxCut}\in R^{+}$ is a parameter set by the user; the second, named starterTriangles, is a subset of the previous one and includes the triangles that have one edge in common with the starting *convex hull*. Therefore, the second list consists of those triangles that should be potentially excluded from the triangulation to improve the definition of the hull. Once the two lists are initialized, the algorithm (whose steps are detailed in Box 4) iterates over the elements of the starterTriangles. In the representation of Fig. 8A, the list consists of two triangles: T_1_ and T_2_. In particular, both edges s_1_e_1_ and s_2_e_2_ belong to the current boundary polygon. Thus opposite vertices p_i_ (*i* = 1,2) are tested in order to be tagged as boundary nodes and to be inserted in the *concave hull*, i.e. the algorithm verifies that the trial boundary edges s_i_p_i_ and p_i_e_i_ do not intersect any edge of the current boundary polygon and that no internal points fall in the triangle s_i_p_i_e_i_. If the admissibility test is passed, T_1_ and T_2_ are removed and the boundary polygon updated (Fig. 8B). Eventually, we go through the triangles that have a side in common with the removed triangles exploiting the neighborlist (see Table 1). If they belong to the outlierTriangles list, they too are added to the starterTriangles list. In the example in Fig. 8B, triangle T_3_ is added.

The algorithm continues to update the starterTriangles list until it is empty. The resulting boundary polygon will then be the final *concave hull*.

The methodology involves a second triangulation on the plane where the *Planar Straight Line Graph* defined by the identified polygon is imposed as a constraint.

The corresponding string used to run the *Triangle* library is given in Box 5.

At last, the constrained triangulation is re-projected in the 3D space to obtain the reconstructed surface (Box 6).

The resulting triangulated surface inherits the IO structure of the Triangle library, i.e. the triangulateio C-struct, whose pointlist array is updated with the 3D re-projected coordinates. It is observed that during the triangulation process no points are added or deleted thus there is a perfect correspondence between the input and output points of the cloud.

## Method validation

**Fig. 9:**
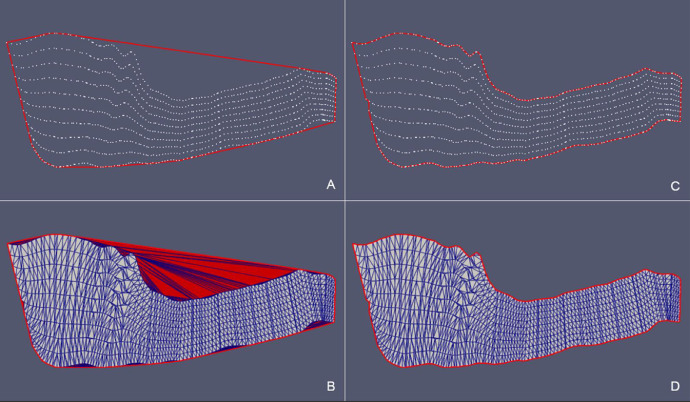
*Fault 12* case – (A) Initial *convex hull* of the point cloud projected on the BFP and (B) corresponding triangulation. (C) *Concave hull* of the point cloud projected on BPF obtianed as the output of the updateHullDig routine with parameter dig = 0.4. (D) Final triangulation.

**Fig. 10:**
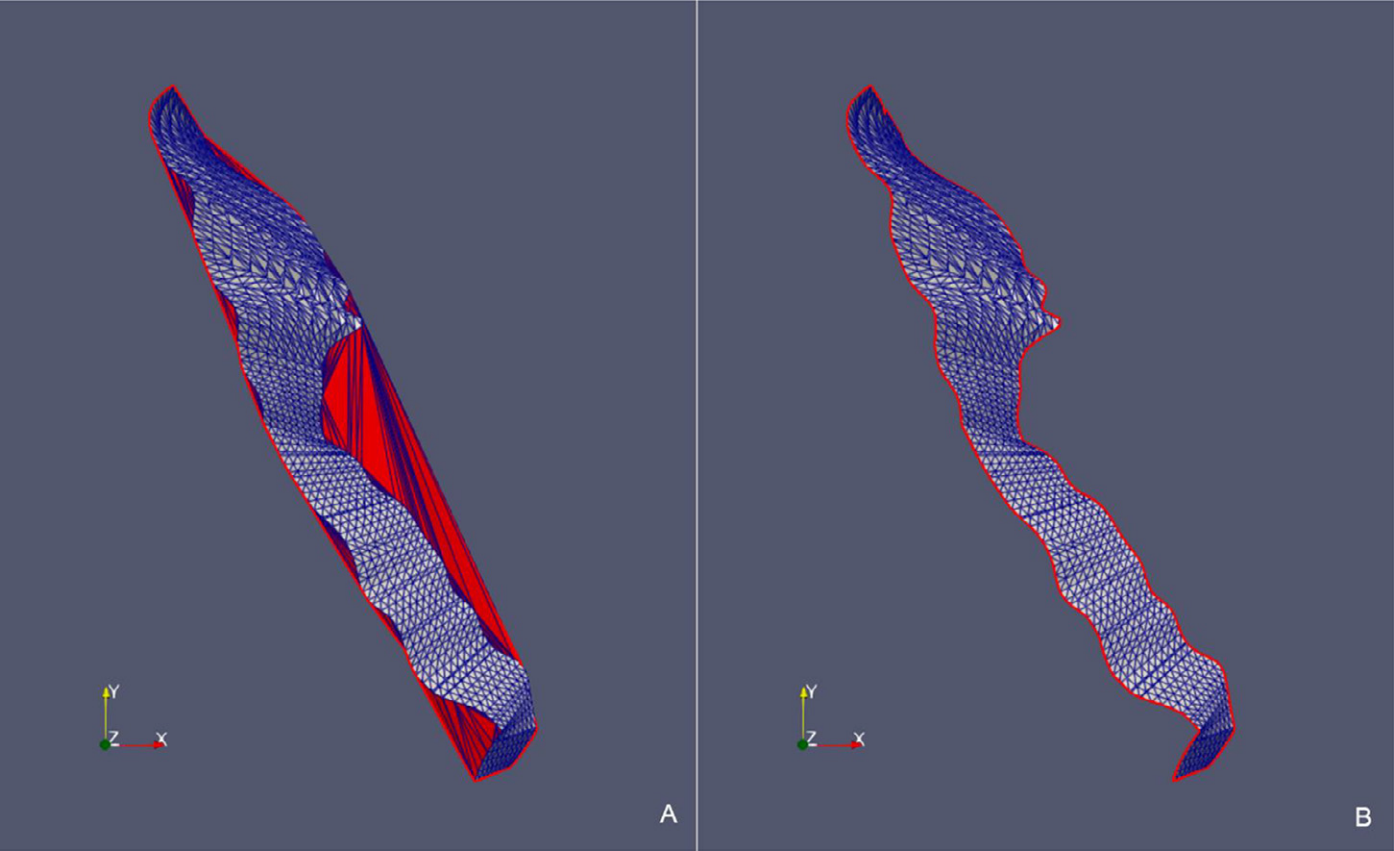
*Fault 12* case – Comparison between the point cloud triangulation constrained to *convex hull* (A) and the triangulation constrained to the *concave hull* (B) in the original xyz Cartesian coordinate system.

**Fig. 11:**
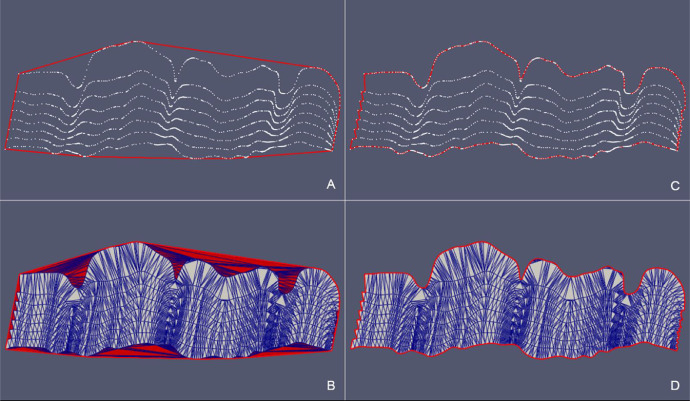
*Fault H53* case – (A) Initial *convex hull* of the point cloud projected on the BFP and (B) corresponding triangulation. (C) *Concave hull* of the point cloud projected on BPF obtianed as the outupt of the updateHullDig routine with parameter dig = 0.75. (D) Final triangulation.

**Fig. 12:**
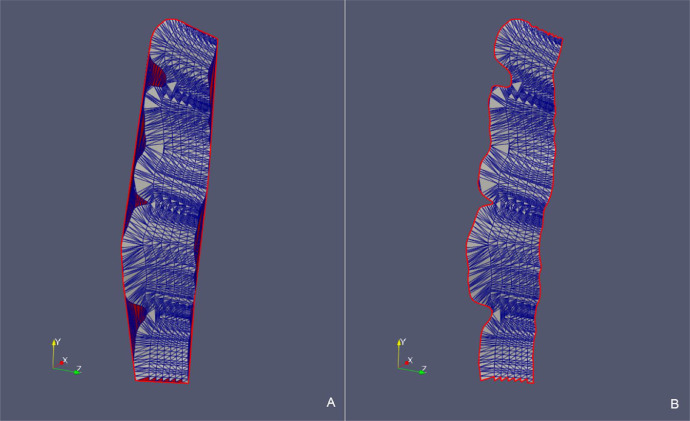
*Fault H53* case – Comparison between the point cloud triangulation constrained to the *convex hull* (A) and the triangulation constrained to the *concave hull* (B) in the original xyz Cartesian coordinate system.

**Fig. 13:**
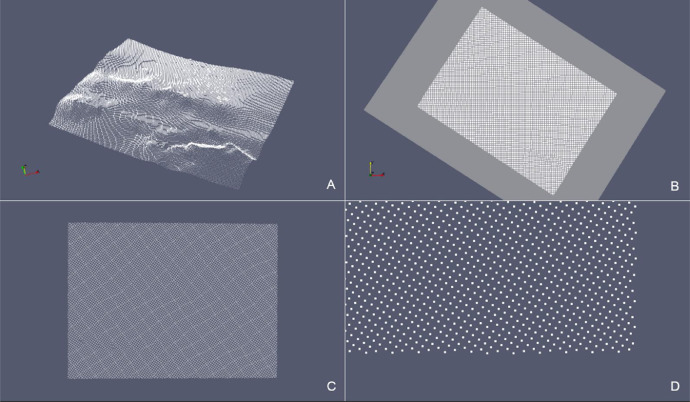
*SurfTop* case – (A) Point cloud in the original xyz Cartesian Coordinate System. (B) Intersection with the xy-plane. (C) Projection of the point cloud onto the xy-plane. (D) Detail of the boundary points.

**Fig. 14:**
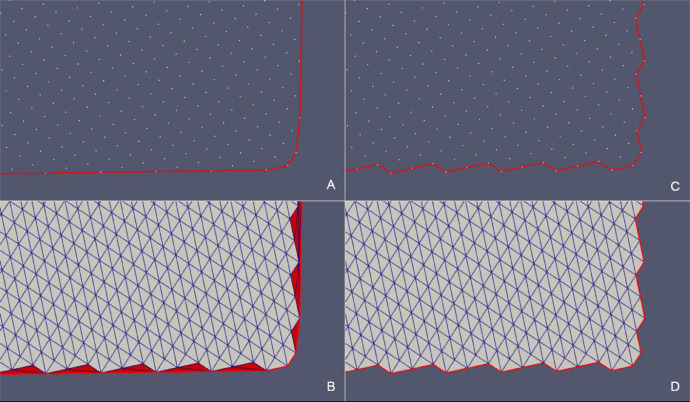
*SurfTop* case – (A) Detail of the starting *convex hull* of the point cloud projected onto the xy-plane and (B) corresponding triangulation (in red triangles that need to be removed). (C) Detail of the *concave hull* of the point cloud projected onto the xy-plane obtianed as the outupt of the updateHullWipe with parameter maxCut = 0.5. (D) Final triangulation.

**Fig. 15:**
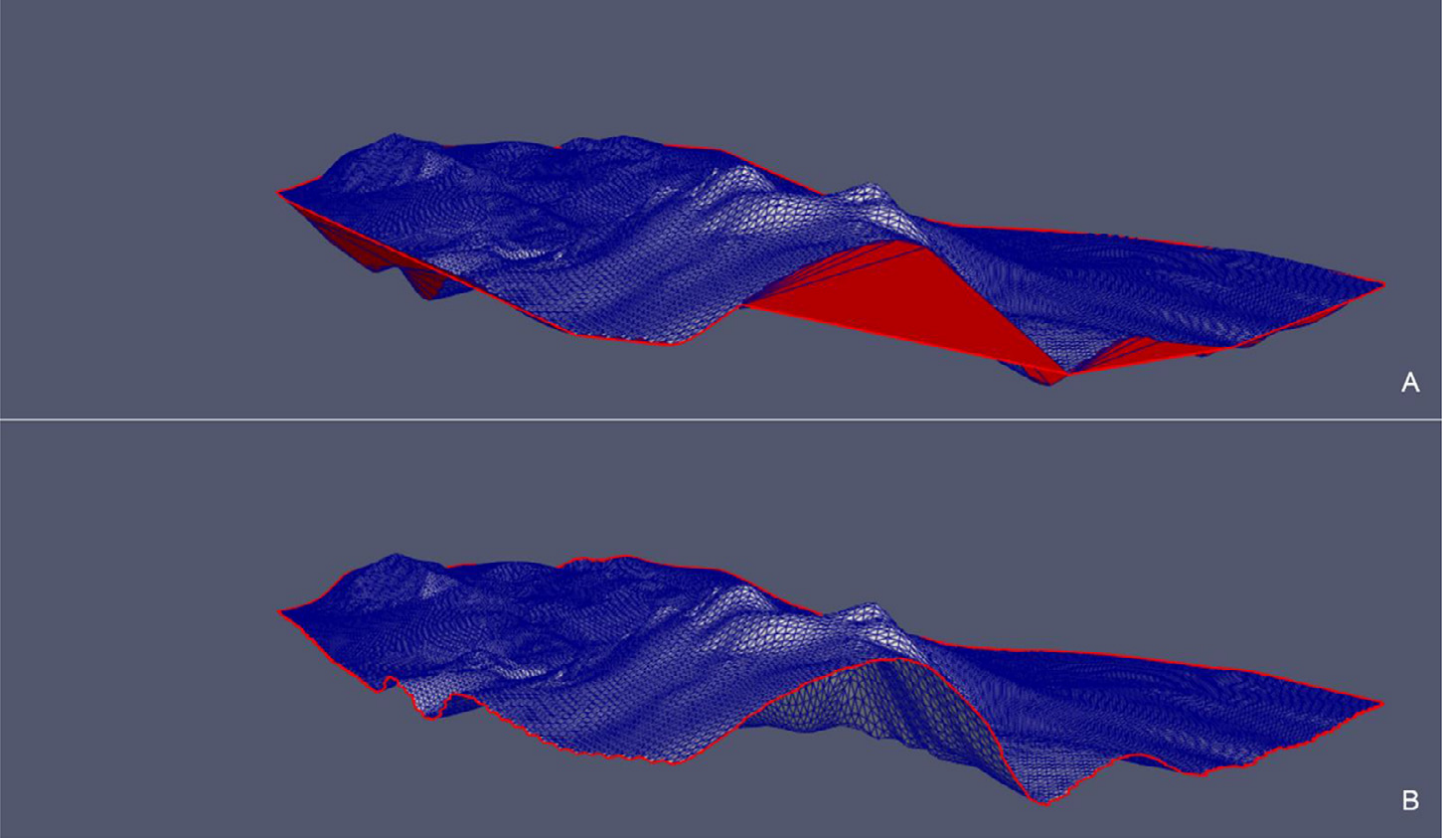
*Surf Top* case – Comparison between the point cloud triangulation constrained to *convex hull* (A) and the triangulation constrained to the *concave hull* (B) in the original xyz Cartesian coordinate system.

**Fig. 16:**
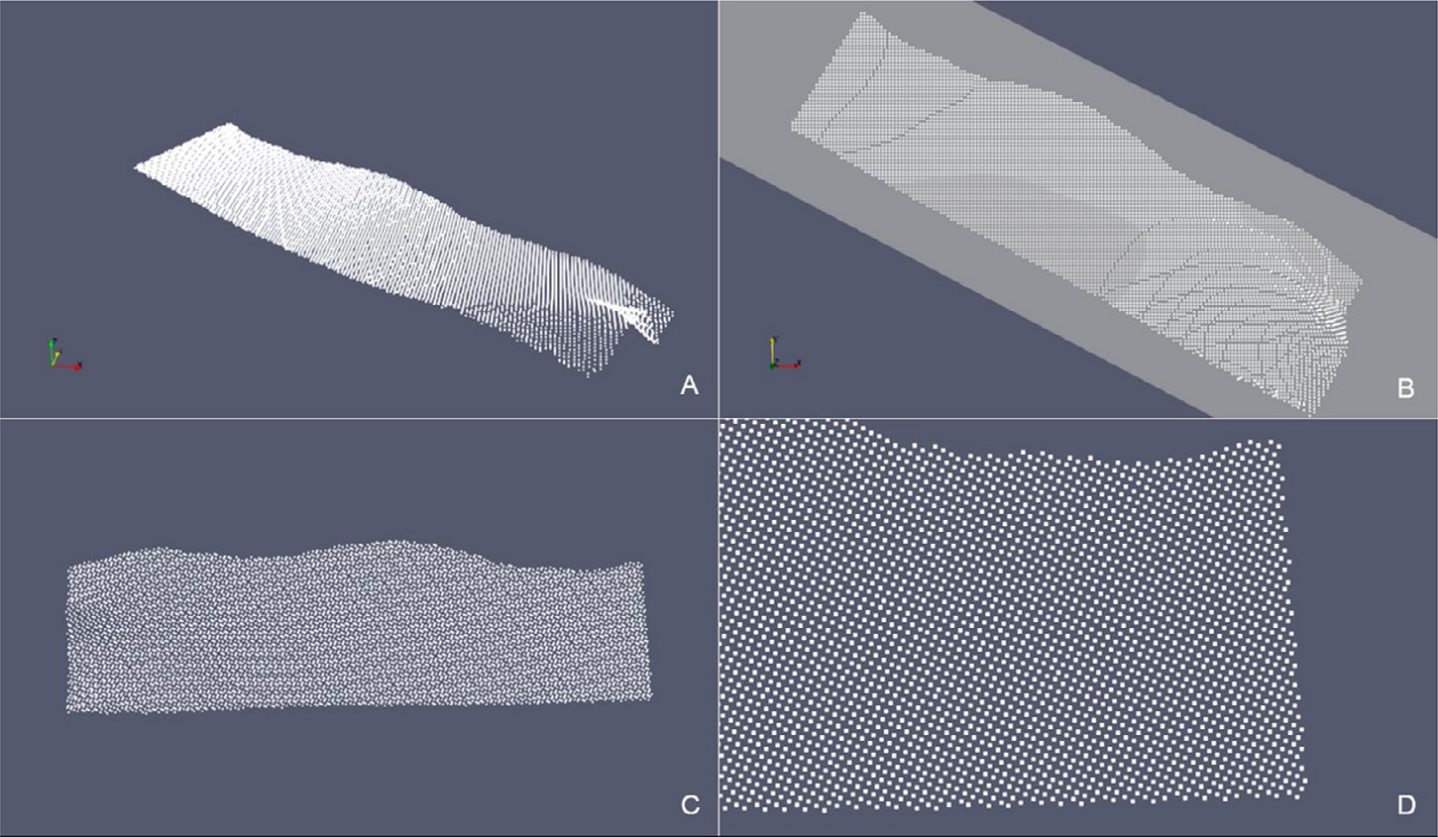
*Erosional 50* case – (A) Point cloud in the original xyz Cartesian Coordinate System. (B) Intersection with the xy-plane. (C) Projection of the point cloud onto the xy-plane. (D) Detail of the boundary points.

**Fig. 17:**
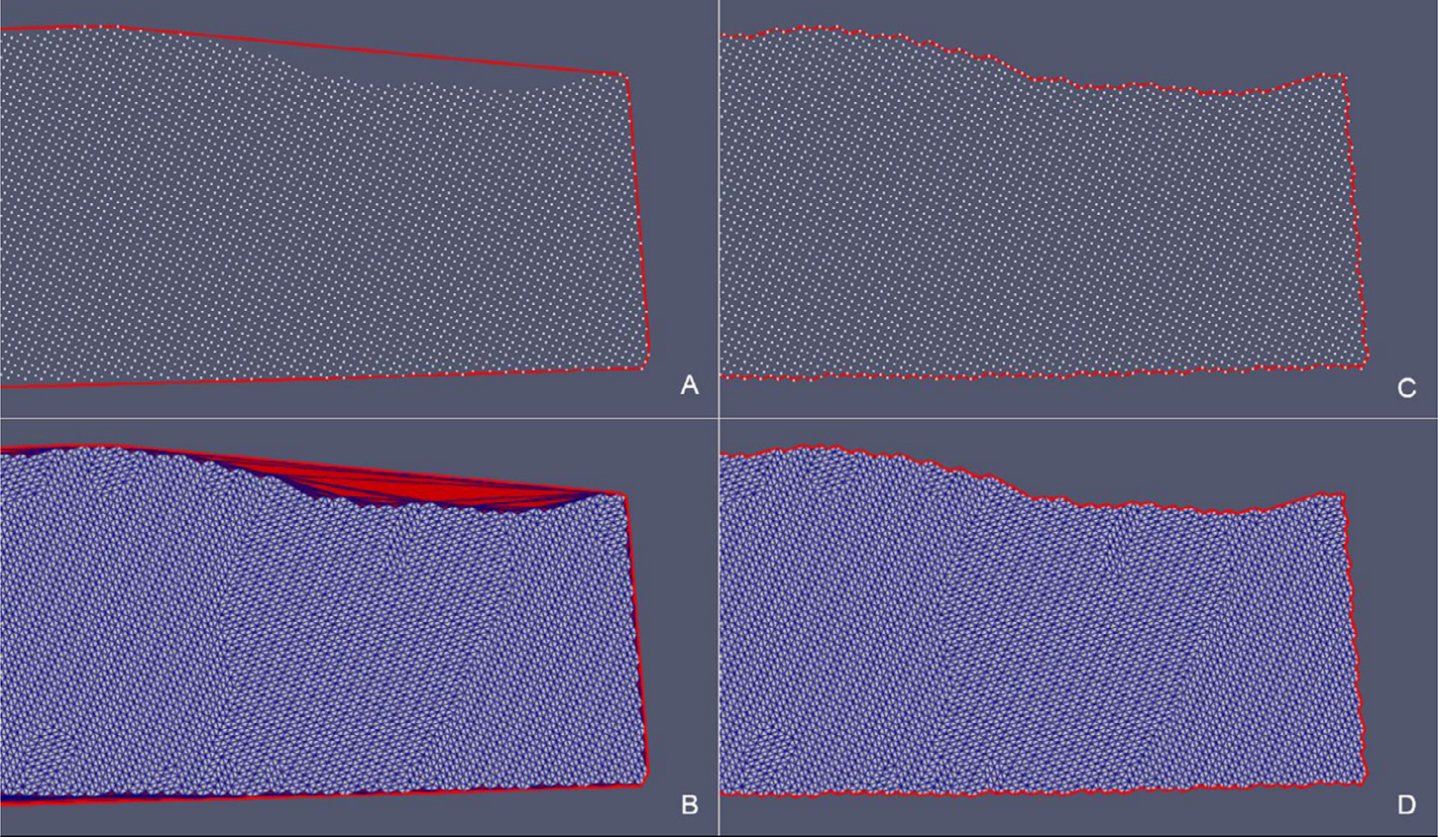
*Erosional 50* case – (A) Detail of the starting *convex hull* of the point cloud projected onto the xy-plane and (B) corresponding triangulation (in red triangles that need to be removed). Detail of the *concave hull* of the point cloud projected onto the xy-plane obtianed as the outupt of the updateHullWipe with parameter maxCut = 0.5 (C). Final triangulation (D).

**Fig. 18:**
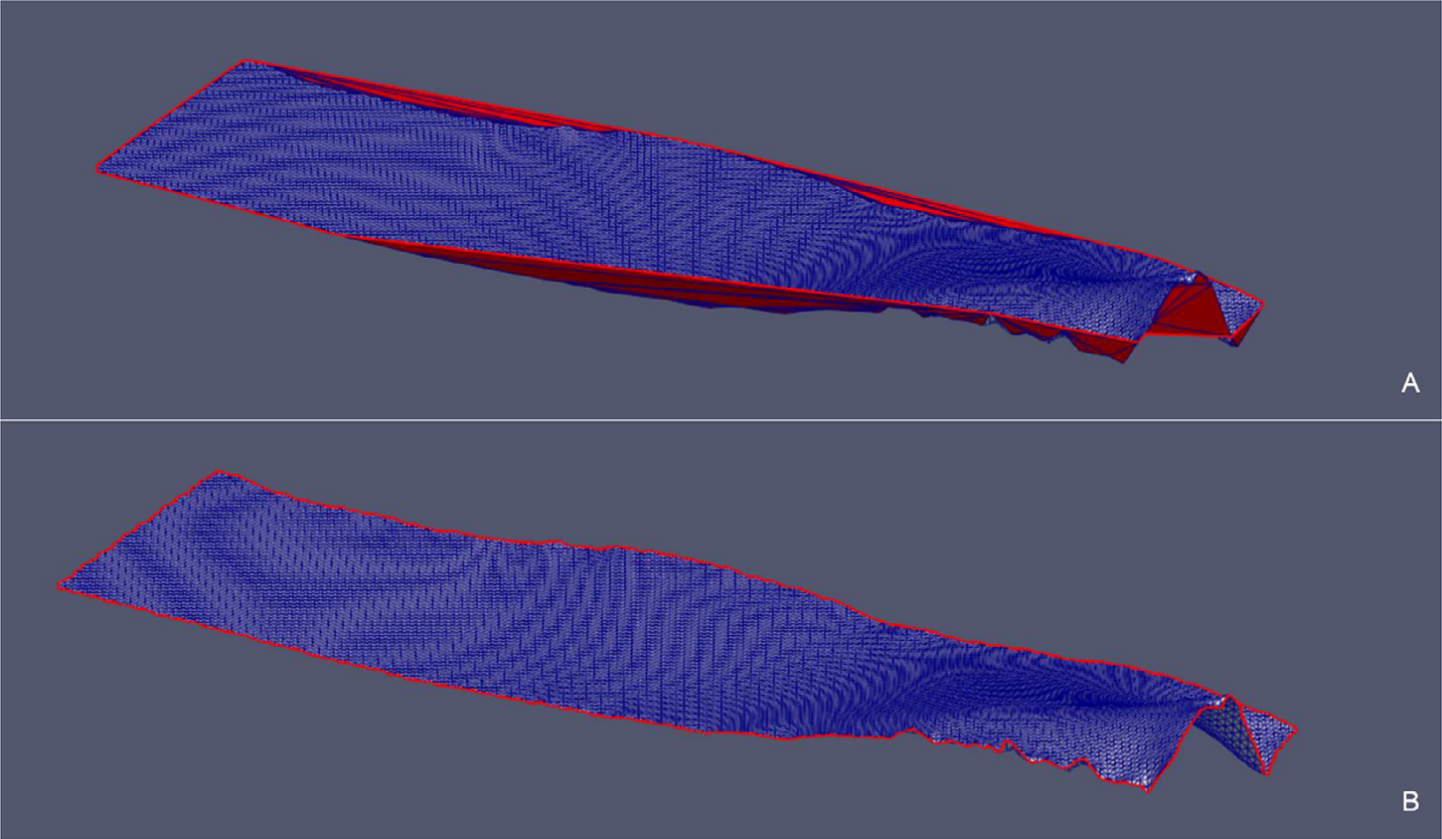
*Erosional 50* case – Comparison between the point cloud triangulation constrained to *convex hull* (A) and the triangulation constrained to the *concave hull* (B) in the original xyz Cartesian coordinate system.

In order to illustrate the effectiveness of the presented method, the reconstruction processes of 2 fault surfaces and 2 stratigraphic surfaces are reported below. Table 2 shows the details of the application of the updateHullDig routine to the cases named *Fault 12* (Fig. 9, Fig. 10) and *H53* (Fig. 11, Fig. 12). In Table 3 we refer to the cases called *Surf Top* (Fig. 13, Fig. 14and Fig. 15) and *Erosional 50* (Fig. 16, Fig. 17and Fig. 18) where the updateHullWipe routine was applied.

**Table 2:** Reconstruction of fault surfaces with the updateHullDig routine. The number of the points in the cloud, the values assigned to the dig parameter, the characteristics of the triangulation referred to the initial *convex hull* and the values relative to the final *concave hull* are given.

Case	# Points	Parameter	Convex Hull		Concave Hull	
		dig	*# Nodes*	*# Triangles*	*# Nodes*	*# Triangles*
*Fault 12*	1110	0.4	40	2178	216	2002
*H53*	1632	0.75	32	3230	436	2826

**Table 3:** Reconstruction of stratigraphic surfaces with the updateHullWipe routine. The number of the cloud points and its sampling increment, the values assigned to the maxCut parameter, the characteristics of the triangulation referred to the initial *convex hull* and the values relative to the final *concave hull* are reported.

Case	# Points	Sampling	Parameter	Convex Hull		Concave Hull	
		[m]	maxCut	*# Nodes*	*# Triangles*	*# Nodes*	*# Triangles*
*Surf Top*	34,635	150 × 150	0.5	32	69,236	627	68,641
*Erosional 50*	7335	50 × 50	0.5	23	14,645	353	14,315

**Box 1:** Functions for deriving the BFP e consequent projection. The points are saved in the Eigen::MatrixXd xyz and their projection on BFP in xyProj.

**Box 2:** Triangle syntax for triangulation of the 2D point cloud without boundary constraints (see details on Triangle switches in Supplementary Material section).

**Box 3:** hull::updateHullDig(double dig) - pseudo-code of the function for the construction of the *concave hull* using the “digging” strategy.

**Box 4:** hull::updateHullWipe(triInfo t, double maxCut) – pseudo-code of the function for the construction of the *concave hull* on the plane. The strategy eliminates triangles by evaluating the length of their edges and whether they belong to the boundary polygon.

**Box 5:** Triangle syntax for the triangulation of the 2D point cloud constrained to the Planar Straight Line Graph (see details on Triangle switches in Supplementary Material section).

**Box 6:** Function for the transformation to the original reference system from local coordinates on the plane.

We observe that in all the presented cases the triangles of the initial triangulation (constrained to the *convex hull*) that generated erroneous “folds” (in red) are correctly removed from the final triangulation (constrained to the *concave hull*). Removed triangles originally connected points that are classified as boundary nodes in the final hull.

## Declaration of Competing Interest

The authors declare that they have no known competing financial interests or personal relationships that could have appeared to influence the work reported in this paper.
