# Supplementary material for: Geological surface reconstruction from 3D point clouds
Source: MethodsX. 2021 May 26;8:101398. doi: 10.1016/j.mex.2021.101398 (PMC8374526; doi:10.1016/j.mex.2021.101398)
Supplement: Supplementary file 1 [file mmc1.pdf]

Supplementary material *and* Additional information:

Triangle Library - switches:

-z: Numbers all items starting from zero (rather than one). Note that this switch is normally overrided by the value used to number the first vertex of the input .node or .poly file.

-n: Outputs (to a .neigh file) a list of triangles neighboring each triangle.

-Y: Prohibits the insertion of Steiner points on the mesh boundary. If specified twice (-YY), it prohibits the insertion of Steiner points on any segment, including internal segments.

-c: Encloses the convex hull with segments.

-p: Triangulates a Planar Straight Line Graph (.poly file).

-e: Outputs (to an .edge file) a list of edges of the triangulation.

For further information refer to the online Triangle Library Manual (https://www.cs.cmu.edu/~quake/triangle.html)

Triangle Library - triangulateio struct:

List of available data

pointlist: An array of point coordinates. The first point's x coordinate is at index [0] and its y coordinate at index [1], followed by the coordinates of the remaining points. Each point occupies two REALs.

pointattributelist: An array of point attributes. Each point's attributes occupy numberofpointattributes REALs.

pointmarkerlist: An array of point markers; one int per point.

trianglelist: An array of triangle corners. The first triangle's first corner is at index [0], followed by its other two corners in counterclockwise order, followed by any other nodes if the triangle represents a nonlinear element. Each triangle occupies numberofcorners' ints.

triangleattributelist: An array of triangle attributes. Each triangle's attributes occupy numberoftriangleattributes REALs.

trianglearealist: An array of triangle area constraints; one REAL per triangle. Input only.

neighborlist: An array of triangle neighbors; three ints per triangle. Output only.

segmentlist: An array of segment endpoints. The first segment's endpoints are at indices [0] and [1], followed by the remaining segments. Two ints per segment.

segmentmarkerlist: An array of segment markers; one int per segment.

holelist: An array of holes. The first hole's x and y coordinates are at indices [0] and [1], followed by the remaining holes. Two REALs per hole. Input only, although the pointer is copied to the output structure for your convenience.

regionlist: An array of regional attributes and area constraints. The first constraint's x and y coordinates are at indices [0] and [1], followed by the regional attribute at index [2], followed by the maximum area at index [3], followed by the remaining area constraints. Four REALs per area constraint. Note that each regional attribute is used only if you select the `A' switch, and each area constraint is used only if you select the `a' switch (with no number following), but omitting one of these switches does not change the memory layout. Input only, although the pointer is copied to the output structure for your convenience.

edgelist: An array of edge endpoints. The first edge's endpoints are at indices [0] and [1], followed by the remaining edges. Two ints per edge. Output only.

edgemarkerlist: An array of edge markers; one int per edge. Output only.

normlist: An array of normal vectors, used for infinite rays in Voronoi diagrams. The first normal vector's x and y magnitudes are at indices [0] and [1], followed by the remaining vectors. For each finite edge in a Voronoi diagram, the normal vector written is the zero vector. Two REALs per edge. Output only.

For further information refer to the online Triangle Library Manual Triangle (https://www.cs.cmu.edu/~quake/triangle.html)

Box 7: hull::getNearestInnerPoint – routine for the research of neighborhood points that are candidates to be tagged as boundary nodes. Constants are defined as MIN_COS = $cos(\pi/2)$ *e EXT_MIN_COS =* $cos(\pi/6)$*.*

```
int hull::getNearestInnerPoint(const int iP1, const int iP2, vector<int>::const_iterator iterP1,
                               const double r)
{
        double dNeigh = r;
        int iNeigh = -1;
        double dNeigh1, dNeigh2;
        const point p1 = pts[iP1];
        const point p2 = pts[iP2];
        int d = 0;

        for (int iNeighTmp = 0 ; iNeighTmp < pts.size(); iNeighTmp++)
        {
                point neighTmp = pts[iNeighTmp];
                dNeigh1 = p1.Distance(neighTmp);
                dNeigh2 = p2.Distance(neighTmp);

                if ( (dNeigh1 <= dNeigh || distNeigh1 <= dNeigh) &&
                     (cosAngleBetweenSegments(p1, neighTmp, p2) >= MIN_COS &&
                      cosAngleBetweenSegments(p2, neighTmp, p1) >=MIN_COS))
                {
                        if (isTriangleEmpty(p1, p2, neighTmp) &&
                           !checkHullEdgeIntersection(p1, p2, neightmp))
                        {
                                point p0, p3;
                                getNeighbourPoints(iterP1, p0, p3);

                                if (cosAngleBetweenSegments(p1, neighTtmp, p0) <= EXT_MIN_COS &&
                                    cosAngleBetweenSegments(p2, neighTmp, p3) <= EXT_MIN_COS)
                                {
                                        dNeigh = min<double>(dNeigh1,dNeigh2);
                                        iNeigh = i;
                                }
                        }
                }
                i++;
        }

return(iNeigh);

}
```

Box 8: `hull::getNeighbourPoints` *- routine or the identification of nodes adjacent to the analyzed segment* p_1p_2*, i.e.* p_0p_1 *e* p_2p_3

```
void hull::getNeighbourPoints(const vector<int>::const_iterator iterP1, point& p0, point& p3)
{
        p0 = (iterP1 != concaveHull.begin()) ? pts[*(iterP1 - 1)] : pts[*(concaveHull.end() - 1)];
        const vector<int>::const_iterator pos2 = pos1 + 1;
        if (pos2 == concaveHull.end())
                p3 = pts[*(concaveHull.begin() + 1)];
        else
                p3 = (pos2 + 1 != concaveHull.end()) ? pts[*(pos2 + 1)] : pts[*concaveHull.begin()];
}
```

Box 9: `hull::cosAngleBetweenSegments` *– routine for the calculation of the cosine of the angle define by segments p1p2 e p2p3. Value –DBL_MAX is equivalent to* $-\infty$*.*

```
inline double hull::cosAngleBetweenSegments(const point p1, const point p2, const point p3)
{
        double d21x = p2.x - p1.x;
        double d31x = p3.x - p1.x;
        double d21y = p2.y - p1.y;
        double d31y = p3.y - p1.y;
        double n21 = sqrt(d21x*d21x + d21y * d21y);
        double n31 = sqrt(d31x*d31x + d31y * d31y);
        return (n21*n31) ? (d21x * d31x + d21y * d31y) / (n21*n31) : -DBL_MAX;
```

Box 10: `hull::isTriangleEmpty` *– function applied to verify that any point of the cloud falls inside or on the edge of the tested triangle (p1 p2 v). If not, it means that the vertex v cannot be tagged as a boundary node.*

```
bool hull::isTriangleEmpty(const point p1, const point p2, const point v)
{
        double sinAngle = sinAngleBetweenSegments(p1, p2, v);
        if (sinAngle == -DBL_MAX)

                return false;
        else
        {
                polygon triangle
                if (sinAngle > 0)
                        triangle.setPolygon(vector<point>{ p1, v, p2 });
                else
                        triangle.setPolygon(vector<point>{ p1, p2, v });

                for (int iPoint = 0 ; iPoint < pts.size(); iPoint++)

                {
                        point p = pts[ip];

                        if (find(triangle.begin(),triangle.end(), p) == triangle.end() &&
                           (triangle.isPointOnThePolygonBorder(p) || triangle.isPointInsidePolygon(p)))
                                return false;
                }

        }

return true;

}
```

Box 11: `hull::sinAngleBetweenSegments` *– routine for the calculation of the sine of the angle define by segments p1p2 e p2p3. Value –DBL_MAX is equivalent to -∞.*

```
inline double hull::sinAngleBetweenSegments(const point p1, const point p2, const point p3)
{
        double d21x = p2.x - p1.x;
        double d31x = p3.x - p1.x;
        double d21y = p2.y - p1.y;
        double d31y = p3.y - p1.y;
        double n21 = sqrt(d21x*d21x + d21y * d21y);
        double n31 = sqrt(d31x*d31x + d31y * d31y);
        return (n21*n31) ? (d21x * d31y - d21y * d31x) / (n21*n31) : -DBL_MAX;
}
```

Box 12: `hull::checkHullEdgeIntersection` *– routine to verify that the new added point (pNew) does not originate segments that intersect the edges of the current hull.*

```
bool hull::checkHullEdgeIntersection(const point edgeStart, const point edgeEnd, const point pNew)
{
	const vector<int>::const_iterator endConcaveHull = concaveHull.end();
	const vector<int>::const_iterator beginConcaveHull = concaveHull.begin();

	for (vector<int>::const_iterator hullPtr = beginConcaveHull; hullPtr != endConcaveHull)
	{
		const point p1 = pts[*hullPtr++];
		point p2;

		if (hullPtr != endConcaveHull)

			p2 = pts[*hullPtr];
		else
			p2 = pts[*beginConcaveHull];


		if (edgeStart == p1 || edgeStart == p2 || edgeEnd == p1 || edgeEnd == p2) continue;

		if (checkEdgeIntersection(edgeStart, pNew, p1, p2)
		    || checkEdgeIntersection(pNew, edgeEnd, p1, p2))  return true;
	}

	return false;

}
```

Box 13: `polygon::isPointInsidePolygon` *– routine to verify whether the tested point (v) falls within a generic polygon. The code is taken form Haines' ptinpoly.cpp published in Graphics Gems IV, Elsevier, 1994,* https://doi.org/10.1016/C2013-0-07360-4

```
bool polygon::isPointInsidePolygon(point v)
{
	bool yFlag0, yFlag1, xFlag0;
	bool insideFlag = false;
	int j = (int)polygon.size() - 1;

	yFlag0 = (polygon[j].y >= v.y);

	for (int i = 0; i < polygon.size(); i++)
	{
		yFlag1 = (polygon[i].y >= v.y);

		if (yFlag0 != yFlag1)
		{
			xFlag0 = (polygon[j].x >= v.x);

			if (xFlag0 == (polygon[i].x >= v.x))
			{
				if (xFlag0) insideFlag = !insideFlag;
			}
			else
			{
				if ((polygon[i].x + (v.y - polygon[i].y) *
				    (polygon[j].x - polygon[i].x)/ (polygon[j].y - polygon[i].y)) >= v.x)
				{
							insideFlag = ! insideFlag;

				}
			}
		}

		yFlag0 = yFlag1;

		j = i;
	}

	return(insideFlag);
}
```

Box 14: `polygon::isPointOnThePolygonBorder` *– routine to verify whether the tested point (v) does not lie on any edge of the current hull*

```
bool polygon::isPointOnThePolygonBorder(point v)
{
        int j = (int)polygon.size() - 1;
        for (int i = 0; i < polygon.size(); i++)
        {
                if (v.isPointOnTheSegment(polygon[j], polygon[i])) return true;
                else j = i;
        }

        return false;
}
```

Box 15: `point::isPointOnTheSegment` *– routine to verify whether the tested point lies on the segment p1p2.*

```
bool point::isPointOnTheSegment(const point p1, const point p2)
{
        double x1 = p1.x;
        double y1 = p1.y;
        double x2 = p2.x;
        double y2 = p2.y;
        int sgn12 = sgn<double>(x2 - x1);
        if (  (abs((y2 - y1)*(x - x1) - (x2 - x1)*(y - y1)) <= 1.0e-14 * (x2 - x1)* (x - x1)
                && (x -x1)*sgn12 > 0 && (x - x1)*sgn12 < abs(x2 - x1)) ) return true;
        else return false;
}
```

Box 16: checkEdgeIntersection – routine to verify whether the two segments p1p2 and p3p4 intersect

```
inline bool checkEdgeIntersection(const point p0, const point p1, point p2, const point p3)
{
        double s1_x = p1.x - p0.x;
        double s1_y = p1.y - p0.y;
        double s2_x = p3.x - p2.x;
        double s2_y = p3.y - p2.y;
        double den = (-s2_x * s1_y + s1_x * s2_y);

        double s1_x_min = min<double>(p0.x, p1.x);
        double s1_x_max = max<double>(p0.x, p1.x);
        double s2_x_min = min<double>(p2.x, p3.x);
        double s2_x_max = max<double>(p2.x, p3.x);

        if (den == 0)
                return s1_x_max > s2_x_min && s1_x_min < s2_x_max && (p2.y - p0.y)*s1_x == s1_y* (p2.x -
        p0.x);

        int sgn_den = sgn<double>(den);
        double s = (-s1_y * (p0.x - p2.x) + s1_x * (p0.y - p2.y));
        double t = (s2_x * (p0.y - p2.y) - s2_y * (p0.x - p2.x));
        return (s * sgn_den > 0 && s * sgn_den < abs(den) && t * sgn_den> 0 && t * sgn_den < abs(den));
```

Box 17: `hull::computeEdgeStats` *– routine dedicated to the calculation of the mean and standard deviation of the edge length distribution. Triangles with at least one edge belonging to the hull are also identified.*

```
triInfo hull::computeEdgeStats()
{
  const int nTriangle = trianglelist.size()/3; if (!nTriangle) return;
  triInfo t; t.edgeLength = vector<double>(3*nTriangle);
  t.triangleOutsidePolygon = vector<bool>(nTriangle); t.edgeOnPolygon = vector<bool>(3*nTriangle);
   t.mu = 0;  t.sigma = 0;
  const vector<int>::iterator endHull = concaveHull.end();
  const vector<int>::iterator startHull = concaveHull.begin();
  vector<bool>::iterator it = t.triangleOutsidePolygon.begin();
  vector<bool>::iterator edgeIt = t.edgeOnPolygon.begin();
  vector<double>::iterator edgeLengthIt = t.edgeLength.begin();
  const vector<bool>::iterator endIt = t.triangleOutsidePolygon.end();
  bool p[3], edgeOnPolygon; int i[3], e[2]; float edgeWeight[3]; double squareEdgeLength = 0;
  double edgeNum = 0; vector<point> v = vector<point>(3); double muSquare = 0;
  int j2; vector<int>::iterator it0, it1;
  int k = 0;
  while (it != endIt)
  {
        i[0] = trianglelist[k++]; i[1] = trianglelist[k++]; i[2] = trianglelist[k++];
        p[0] = pointmarkerlist[i[0]]; p[1] = pointmarkerlist[i[1]]; p[2] = pointmarkerlist[i[2]];
        // VERTICES OF THE TRIANGLE
        v[0] = point(pointlist[2 * i[0]], pointlist[2 * i[0] + 1]);
        v[1] = point(pointlist[2 * i[1]], pointlist[2 * i[1] + 1]);
        v[2] = point(pointlist[2 * i[2]], pointlist[2 * i[2] + 1]);
        if ((*it++ = p[0] & p[1] & p[2]))  { edgeLengthIt += 3; edgeIt += 3; }
        else
        {
                edgeOnPolygon = true;
                if (p[0] + p[1] + p[2] == 2)
                {
                        edgeOnPolygon = false;
                        for (int j1 = 0; j1 < 3; j1++)
                                if (!p[j1])
                                {
                                        e[0] = i[(j1 + 1) % 3]; e[1] = i[(j1 + 2) % 3];
                                        it0 = find(startHull, endHull, e[0]);
                                        it1 = find(startHull, endHull, e[1]);
                                        if (it0 != startHull & it0 != endHull - 1)
                                                edgeOnPolygon = it0 + 1 == it1 || it0 - 1 == it1;
                                        if (it0 == startHull)
                                                edgeOnPolygon = it1 == endHull - 1;
                                        if (it0 == endHull - 1)
                                                edgeOnPolygon = it1 == startHull;
                                        if (edgeOnPolygon)
        edgeOnPolygon = !checkHullEdgeIntersection(v[(j1 + 1) % 3], v[(j1 + 2) % 3], v[j1]);
                                }
                                *it = !edgeOnPolygon;
                }
                if (edgeOnPolygon)
                        for (int j1 = 0; j1 < 3; j1++)
                        {
                                j2 = (j1 + 1) % 3;
                                *edgeIt = p[j1] & p[j2];
                                edgeWeight[j1] = (*edgeIt++) ? 1 : 0.5;
                                edgeNum += edgeWeight[j1];
                        squareEdgeLength = (pow(pointlist[2 * i[j1]] - pointlist[2 * i[j2]], 2) +
                                            pow(pointlist[2 * i[j1] + 1] - pointlist[2 * i[j2] + 1], 2));
                                muSquare += squareEdgeLength * edgeWeight[j1];
                                *edgeLengthIt = sqrt(squareEdgeLength);
                                t.mu += *edgeLengthIt++*edgeWeight[j1];
                        }
                else { edgeLengthIt += 3; edgeIt += 3; }
        }
  }
  if (edgeNum)
  {
        t.mu /= edgeNum; muSquare /= edgeNum;
        t.sigma = sqrt(edgeNum / (edgeNum - 1)*(muSquare - triInfo.mu*triInfo.mu));
  }
  else { t.mu = -DBL_MAX; t.sigma = -DBL_MAX; }
}
```

Future Development:

The neighborhood exploration on the BFP projection represents a bottleneck of the algorithm with the increasing dimension of the point cloud, especially in the case of stratigraphic surfaces that can have the same areal extension of the whole model. An optimization of the algorithm could take into account a parametrization of the search step, i.e. a rectangle R could be defined using the bounding values [xmin xmax, ymin ymax] of the projected point cloud. The rectangle R encloses, by definition, all the 2D points. Thus R is divided in N sub-rectangles r_k (0≤k≤N), and each projected point is mapped to the corresponding k-value. In this way, the search for the new vertex of the boundary polygon is reduced to a portion of the 2D domain, i.e. it is focused to the subset of r_k which encloses the neighborhood. Such a strategy reduces the computational cost of the algorithm and it could be parallelized by assigning portions of the domain to different threads.
